# Chemical Stability of Ascorbic Acid Integrated into Commercial Products: A Review on Bioactivity and Delivery Technology

**DOI:** 10.3390/antiox11010153

**Published:** 2022-01-13

**Authors:** Xin Yin, Kaiwen Chen, Hao Cheng, Xing Chen, Shuai Feng, Yuanda Song, Li Liang

**Affiliations:** 1 State Key Laboratory of Food Science and Technology, Jiangnan University, Wuxi 214122, China; 17851312787@163.com (X.Y.); chenkaiwen1018@163.com (K.C.); haocheng@jiangnan.edu.cn (H.C.); xingchen@jiangnan.edu.cn (X.C.); 2 School of Food Science and Technology, Jiangnan University, Wuxi 214122, China; 3 Luwei Pharmaceutical Group Co., Ltd., Shuangfeng Industrial Park, Zibo 255195, China; fengshuai@hlvitamin.com; 4 Colin Raledge Center for Microbial Lipids, School of Agricultural Engineering and Food Science, Shandong University of Technology, Zibo 255000, China; ysong@sdut.edu.cn

**Keywords:** ascorbic acid, bioactivity, stability, delivery, application

## Abstract

The L-enantiomer of ascorbic acid is commonly known as vitamin C. It is an indispensable nutrient and plays a key role in retaining the physiological process of humans and animals. L-gulonolactone oxidase, the key enzyme for the de novo synthesis of ascorbic acid, is lacking in some mammals including humans. The functionality of ascorbic acid has prompted the development of foods fortified with this vitamin. As a natural antioxidant, it is expected to protect the sensory and nutritional characteristics of the food. It is thus important to know the degradation of ascorbic acid in the food matrix and its interaction with coexisting components. The biggest challenge in the utilization of ascorbic acid is maintaining its stability and improving its delivery to the active site. The review also includes the current strategies for stabilizing ascorbic acid and the commercial applications of ascorbic acid.

## 1. Introduction

**Figure 1:**
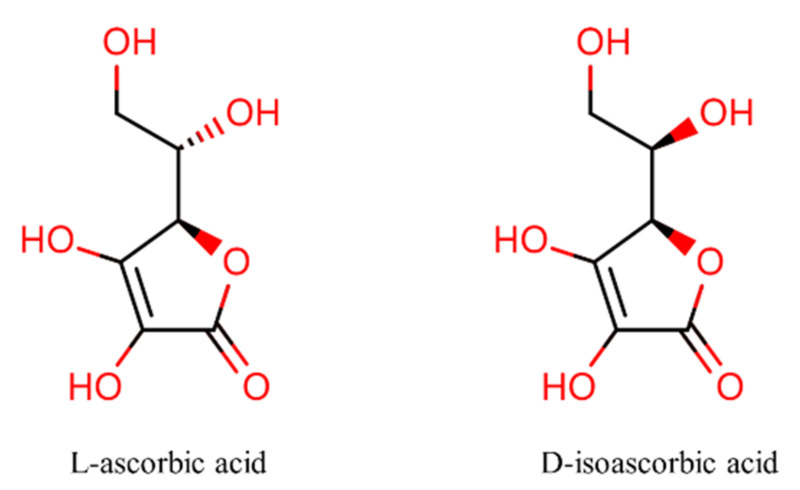
Structures of L-ascorbic acid and its stereoisomer.

Ascorbic acid (L-enantiomer, Figure 1), commonly known as vitamin C, is composed of six carbons and related to the C_6_ sugars. It is the aldono-1,4-lactone of a hexonic acid with an enediol group on carbons 2 and 3. As an essential micronutrient, ascorbic acid plays a vital role in maintaining normal metabolic processes and homeostasis within the human body. Although D-isoascorbic acid is the stereoisomer of ascorbic acid (Figure 1), such analogues hardly express the activity of ascorbic acid. Mammalian cells cannot synthesize ascorbic acid de novo due to the lack of L-gulono-1,4 lactone oxidase, which is an essential enzyme for the production of ascorbic acid [1]. Vegetables and fruits serve as natural sources of vitamin C intake, but only a limited number of plants are rich in vitamin C. Nowadays, ascorbic acid is industrially produced from D-glucose, and the procedure involves several complex chemical and biotechnological stages [2].

An ordinary diet of natural and synthetic ascorbic acid is the only way to maintain the physiological requirements. The well-known symptom of ascorbic acid deficiency is associated with connective tissue damage, such as scurvy, which is characterized by fragile tissues and poor wound healing [3]. The currently recommended dietary allowances (RDA) for ascorbic acid are 90 mg/day and 75 mg/day for men and women, respectively [4]. Researchers have found that the steady-state concentration of ascorbic acid in plasma is about 80 μmol/L, when sufficient fruits and vegetables are consumed every day. Oral dosing of ascorbic acid (1.25 g) can improve the concentration of ascorbic acid in plasma to 134.8 ± 20.6 μmol/L [5]. In order to maintain the ascorbic acid concentration required by the body, ascorbic acid-fortified dietary supplements or foods have attracted interest from consumers, in addition to fruits and vegetables found in nature.

**Figure 2:**
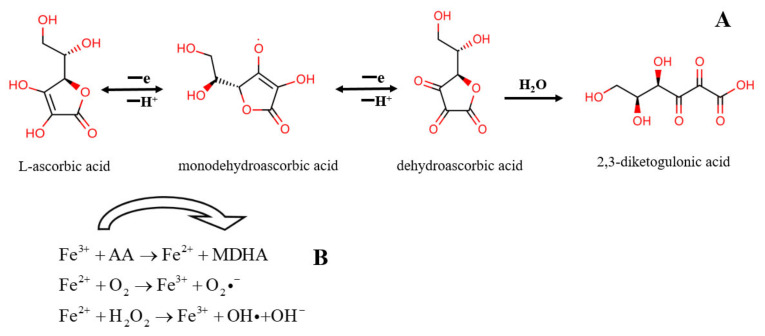
Degradation of L-ascorbic acid to dehydroascorbic acid and 2,3-diketogulonic acid (**A**) and pro-oxidant effects of ascorbic acid (**B**).

The main challenge in the development of ascorbic acid products is its high instability and reactivity. Ascorbic acid is reversibly oxidized into dehydroascorbic acid (DHA) upon exposure to light, heat, transition metal ions and pH (alkaline condition), then DHA further irreversibly hydrolyzes to form 2,3-diketogulonic acid (Figure 2A). In recent decades, the strategy of shielding ascorbic acid from sensitive environments by encapsulating ascorbic acid within a layer of wall material has attracted much interest among researchers. A series of innovation delivery technologies have emerged, including microfluidic [6], melt extrusion [7], spray drying and chilling [8,9]. The particles prepared by these methods are usually on the microscale. Under certain conditions, nano-encapsulation of ascorbic acid can be realized through ion gelation of chitosan or complex coacervation with anionic polymers [10,11]. On the other hand, some bioactive compounds with low molecular weight can protect ascorbic acid by scavenging the pro-degradation factors of ascorbic acid in the solution [12].

Stable ascorbic acid needs to be accurately delivered to the desired site, and released from the carrier at a desired rate and time. This is the basis for obtaining excellent bioavailability of ascorbic acid. For oral products, the expected release site of ascorbic acid is the small intestine rather than the stomach, since the absorption and metabolization of bioactive compounds mainly occur in the small intestine [13]. This is a challenge for the selection of suitable carrier and wall materials, which is related to the dissolution of the coating polymer in the gastrointestinal environment and its molecular weight. There is a hypothesis that the molecular weight of the used polymers is negatively related to the release of the encapsulated compounds [14]. The encapsulation efficiency of ascorbic acid in gelatin-coated microcapsules reached up to about 94%, but the release of ascorbic acid in the stomach was faster than that in the intestine [15]. However, only 30% of ascorbic acid in chitosan nanoparticles was released in a simulated gastric solution, while the release in simulated intestinal condition exceeded 75% [16].

**Figure 3:**
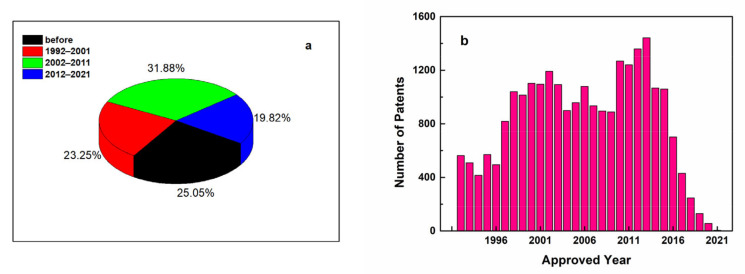
The proportion of patents related to ascorbic acid search terms before 2021 (**a**) and the number of ascorbic acid patents in each of the past 30 years (**b**).

Figure 3 shows the number of patents related to ascorbic acid from 1992 to 2021; there have been a stable and relatively high number of applications since 1997. From 2010 to 2013, the patents were even more than 1200 per year. It can be found that the importance of ascorbic acid has aroused widespread interest in the consumer market. Ascorbic acid itself or in conjunction with co-existing ingredients in the food matrix can express various physiological activities beneficial to health. This review mainly aims to provide a comprehensive summary about the strategies for stabilizing and controlling release of ascorbic acid in the past 20 years. The commercial products fortified with ascorbic acid are also summarized.

## 2. Bioactivity of Ascorbic Acid

### 2.1. Antioxidant

The by-products of normal cell metabolism are reactive oxygen species (ROS), including superoxide radicals (O_2_·^−^), singlet oxygen (^1^O_2_), hydrogen peroxide (H_2_O_2_) and highly reactive hydroxyl radicals (OH·). The adverse effect of ROS is that it can initiate a cascade of radicals, producing hydroxyl free radicals and other destructive species. These further induce protein and DNA damage, lipid peroxidation and finally lead to cell apoptosis [17]. The antioxidant defense system cannot fully eliminate the toxic ROS accumulated in the cells, that is, the so-called “oxidative stress” occurs [18]. In addition to enzymatic reactions, ROS can also be eliminated through non-enzymatic means such as antioxidants. Ascorbic acid is a free radical and other oxygen species scavenger, which can protect cells from oxidative damage caused by ROS. Antiradical capability commonly reflects the antioxidant ability, and ascorbic acid in foodstuff and bio-systems acts as antioxidant. As the most effective and natural antioxidant with the least side effects, ascorbic acid can inhibit various diseases caused by oxidative stress in the body, such as cancer, cardiovascular disease, aging and cataracts [15]. Studies have shown that the mortality from these diseases is inversely related to plasma concentration of ascorbic acid [19]. Ascorbic acid and its derivatives can reduce the level of lipid peroxidation in vivo due to aging [20]. In the absence of transition metals, ascorbic acid can reduce the frequency of mutations induced by H_2_O_2_ in human cells [21]. Compared with other polyphenols or flavonoid antioxidants, ascorbic acid terminates the free radical chain reaction through disproportionation reaction, and the reaction products such as DHA and 2,3-diketogulonic acid (Figure 2A) are non-damaging and non-radical products [22]. Another manifestation of antioxidant property is that ascorbic acid can form relatively stable ascorbic acid free radicals to donate single electrons [23]. As reported, antioxidants can also repair tryptophan free radicals produced by the one-electron oxidation of free tryptophan in lysozyme to maintain protein integrity [24].

Ascorbic acid is also used as an antioxidant to protect the sensory and nutritional properties of foods. As an anti-browning agent, it can inhibit the browning of vegetables and fruits caused by oxidation. The formation of quinones mediated by polyphenol oxidase causes the accumulation of H_2_O_2_, which in turn causes the browning of polyphenols mediated by peroxidase [25]. Ascorbic acid inhibits browning by reducing the o-quinone produced by polyphenol oxidase to the original diphenol through a process called “deactivation reaction” [26]. In addition to the regeneration mechanism of polyphenols, the protective effect is also attributed to the competitive inhibition of polyphenol oxidase activity by ascorbic acid. Meanwhile, addition of ascorbic acid causes a decrease in pH and is not conducive to the expression of polyphenol oxidase activity [27]. In meat products, ascorbic acid is widely used as a natural agent for color retention, which can inhibit lipid oxidation and maintain color stability [28]. Compared with other organic acids such as malic acid, citric acid and tartaric acid, ascorbic acid had the best protective effect on the quality of cured meat and was a suitable ingredient for cured meat products [29]. The surface of the pork sprayed with ascorbic acid and a mix of that and rosemary extract maintained good stability in color, water content and pH after frozen storage [30]. It is worth noting that this dietary source of ascorbic acid added in meat products is often overlooked. Norwegian researchers found that the content of ascorbic acid in sausages is 11–40 mg/mL, but ascorbic acid is usually ignored in the table of food ingredients because the added ascorbic acid is used as a color retention agent rather than a nutrient component. As a result, the actual ascorbic acid intake of Norwegian residents increased by 3–10% [31]. The ascorbic acid added to the edible polysaccharide film can eliminate or quench the free radicals generated by radiation. As a radiation inhibitor, ascorbic acid can inhibit the decrease in the viscosity of carrageenan caused by radiation and protect its rheological properties [32]. Ascorbic acid can inhibit food-borne pathogens in the early stage of biofilm formation due to its anti-quorum sensing activity and inhibition of extracellular polymer production. The efficacy of ascorbic acid is related to its concentration and the strain. For *Escherichia coli* and *Staphylococcus aureus*, the inhibitory effect of ascorbic acid at 25 mg/mL is the greatest, and lower concentrations of ascorbic acid are ineffective. For *Listeria monocytogenes*, ascorbic acid at 0.25 mg/mL shows an inhibitory effect [33].

### 2.2. Pro-Oxidant

Pro-oxidant activity is defined as the ability of antioxidants to reduce transition metal ions to a lower oxidation state, which refers to the Fenton reaction [34]. In the Fenton reaction, transition metal ions such as Fe^3+^ are reduced by ascorbic acid and then Fe^2+^ further react with oxygen and hydrogen peroxide to form highly active and destructive hydroxyl radicals (Figure 2B) [35]. Ascorbic acid does not always express antioxidant activity, and may be converted into a pro-oxidant and show toxic effects under certain conditions. The effect of ascorbic acid on the redox properties of bovine hemoglobin is dual with an antioxidant effect at the initial stage of the reaction. During the reaction process, ascorbic acid generates hydrogen peroxide under the mediation of oxygen or oxygenated hemoglobin. With the consumption of ascorbic acid, its own scavenging ability cannot balance the accumulated hydrogen peroxide, which leads to the formation of bilirubin and accelerates the oxidation of hemoglobin [36]. Studies have shown that ascorbic acid will transform from an antioxidant under physiological conditions to a pro-oxidant at higher concentrations. Researchers found that supplementation of 500 mg of ascorbic acid in the diet for 6 weeks increases the level of oxidative damage to peripheral blood lymphocytes, although this result is still controversial in the academic community [37,38]. Furthermore, the presence of transition metal ions in the system is also a key factor for ascorbic acid exerting pro-oxidant activity [39]. In mayonnaise, the added ascorbic acid works as a lipid antioxidant or pro-oxidant depending on the presence or absence of the fat-soluble antioxidant vitamin E. In the system containing vitamin E, the synergistically antioxidant effect of these two vitamins is stronger than the pro-oxidant effect of ascorbic acid. Without the addition of vitamin E, the hydrogen peroxide at the interface of the oil droplets promotes the lipid oxidation of lipoprotein particles in the mayonnaise, which in turn induces the oxidation of apolipoproteins and produces volatile odors [40]. In addition, dehydroascorbic acid may irreversibly degrade and produce highly reactive carbonyl intermediates, which can induce glycosylation of proteins. This is a non-enzymatic, non-specific reaction between carbonyl and amino groups, which is involved in a variety of age-related diseases [41]. It is worth noting that the pro-oxidation of ascorbic acid can induce the apoptosis of cancer cells, thereby exerting anti-cancer effects to a certain extent. As reported, the copper-dependent cellular redox state is an important factor in the cytotoxic effect of ascorbic acid on cancer cells. Ascorbic acid mobilizes nuclear copper to cause pro-oxidative cleavage of cellular DNA, and nuclear copper serves as a new molecular target for the toxic effects of cancer cells [42,43]. From this perspective, the pro-oxidation effect of ascorbic acid is beneficial.

Up to now, the mechanism and conditions that induce ascorbic acid to express pro-oxidant properties have not been clearly elucidated, and the definition of the conversion concentration between antioxidant and pro-oxidant is also unclear. Moreover, most of the reports on the promotion of oxidation of ascorbic acid are concentrated in vitro [42,43,44].

### 2.3. Co-Factors

Ascorbic acid can also be used as a co-factor for enzymes and other bioactive components to indirectly exert biological activities by acting as a free radical scavenger and electron transfer donor/acceptor to directly express its antioxidant properties. In the metabolic process of animals and plants, ascorbic acid does not directly participate in the catalytic cycle. As an enzyme co-factor, ascorbic acid exerts its indispensable function by regulating hydroxylation processes in multiple enzymatic reactions. For the active part of the enzyme with iron or copper, the role of ascorbic acid is to maintain the transition metal ions of these enzymes in a reduced form to exert their maximum physiological activity [45]. Ascorbic acid is a co-factor for non-heme iron α-ketoglutarate-dependent dioxygenases such as prolyl 4-hydroxylase with the role in the synthesis of collagen. As an electron donor, ascorbic acid can keep iron in the ferrous state, thereby maintaining the full activity of collagen hydroxylase. This promotes the hydroxylation of proline and lysine residues, allowing pro-collagen correct intracellular folding [46]. Ascorbic acid can also promote catecholamine synthesis by circulating tetrahydrobiopterin and enhance adrenal steroid production by increasing the expression of tyrosine hydroxylase [47]. As a co-factor, it helps dopamine β-hydroxylase convert dopamine to norepinephrine [48]. In addition, ascorbic acid can regulate cardiomyopathy and neurometabolic diseases. For example, as a co-factor for carnitine synthesis, it can shuttle fatty acids into the mitochondria and reduce oxidative stress [49]. In some clinical situations, as a co-factor for the biosynthesis of amidated opioid peptides, taking ascorbic acid can exhibit analgesic effects [50].

### 2.4. Synergistic Effect

As a natural antioxidant, ascorbic acid mostly exists in the form of coexistence with other components in nature. Combining it with other antioxidants may produce additive or even synergistic effects. Ascorbic acid and vitamin E, as chain-scission antioxidants, have an important inhibitory effect on the auto-oxidation of cell membrane polyunsaturated liposomes in vivo and the oxidation of lipids in vitro [51]. Studies have shown that the combination of 15% ascorbic acid and 1% α-tocopherol can significantly inhibit erythema and the formation of sunburn cells [52]. The synergy between α-tocopherol and ascorbic acid relies on the ability of ascorbic acid to regenerate α-tocopherol, and maintain the antioxidant capacity of α-tocopherol through circulation and inhibition of pro-oxidation [53]. The combined use of ascorbic acid and gallic acid is a promising strategy to prevent the formation of advanced glycation end products, showing the synergistic effect in the inhibition of amyloid cross-β-structure and protein carbonyl formation in fructose-induced BSA glycosylation samples [54]. Lycopene can inhibit inflammation and further stimulate the release of anti-inflammatory cytokine IL-10 when it combined with ascorbic acid and/or α-tocopherol [55]. Understanding the synergy between ascorbic acid and other bioactive compounds allows the antioxidant system of foods and drugs to be selected more specifically.

## 3. Sensitivity to Environment

### 3.1. Concentration and pH

Ascorbic acid is unstable in aqueous solutions, and its degradation has been considered the main cause of quality and color changes during food storage and processing. Stability analysis of ascorbic acid is a key point in its application. In addition to the interference of external factors, the concentration of ascorbic acid in the solution will also affect its stability. As reported, after storage at room temperature in the presence of light for 27 days, an aqueous solution of 1% concentration of ascorbic acid lost around 21% of its initial concentration, while the 10% ascorbic acid system only degraded about 8% [56]. In order to ensure sufficient content of ascorbic acid in the product, reinforcing the ascorbic acid content is a method commonly used in the food industry. The content of ascorbic acid in fortified milk decreased from 36.4 mg/L to 26.1 mg/L after sterilization, while the ascorbic acid content of normal milk dropped from 12.2 mg/L to 8.3 mg/L. Although the loss content of ascorbic acid increased with the addition of ascorbic acid, the loss efficiency decreased compared with the initial concentration of ascorbic acid. According to the stability and the degradation kinetics of ascorbic acid, a higher concentration of ascorbic acid has a lower degradation rate constant [57]. However, some studies have shown that excessive ascorbic acid (AH_2_) is prone to auto-oxidation to produce dehydroascorbic acid anions, according to the following reaction [58]:

$$AH_{2}\to AH^{-}+H\;AH^{-}+O_{2}\to \cdot A^{-}+O_{2}{}^{-}+H^{+}$$

Furthermore, the autoxidation of ascorbic acid depends on pH. Without transition metal catalysis, its spontaneous oxidation under neutral conditions is quite slow. At pH 7, 99.9% of ascorbic acid is in the form of ascorbate (AH^−^), as well as a small amount of ascorbic acid (AH_2_, ~0.1%) and ascorbate dianion (A^2^^−^, 0.005%) [59].

$$AH_{2}\overset{pK_{a}4.2}{\to }AH^{-}\overset{pK_{a}11.6}{\to }A^{2-}$$

$$A^{2}{}^{-}+O_{2}\to \cdot A^{-}+O_{2}{}^{-}$$

The amount of A^2^^−^ increases 10-fold for every unit increase in pH. Auto-oxidation occurs through A^2^^−^, thus accelerating the oxidation rate of ascorbic acid and hydrolyzing to 2,3-diketogulonic acid in alkaline solution [60]. It is worthy to note that the pH value of the ascorbic acid solution changes with its degradation process. Ascorbic acid with an initial pH of alkaline would degrade to about 7.3, which was affected by degradation products generated from ascorbic acid once the reaction began [61]. In acidic solutions, the degradation products of ascorbic acid are also related to oxygen. Under aerobic conditions, dehydroascorbic acid is further degraded to form 2-furoic acid and 3-hydroxy-pyrone. The main degradation product in anaerobic environment is furfural, and the intermediate product does not involve dehydroascorbic acid [62]. However, in alkaline solutions, the main products of ascorbic acid are 2-methylfuran, 2,4-dimethylfuran, 2-acetyl-5-methylfuran and 2-methyl-2-cyclopentanone [61]. Compared with the hydrolytic pathway that directly cleaves the lactone ring of ascorbic acid under anaerobic conditions, the oxidoreductive pathway in which ascorbic acid forms dehydroascorbic acid in the presence of oxygen is more common in food systems [62]. Although the headspace can be filled with nitrogen, the dissolved oxygen in the solution is difficult to remove. Previous researchers found that there is a linear relationship between the first-order kinetic constant of ascorbic acid degradation in juice and the initial headspace oxygen concentration during storage at 22 °C [63].

### 3.2. Temperature

Ascorbic acid is severely degraded by heat, and the instability of ascorbic acid in thermal-processed foods impedes its application. The degradation of ascorbic acid involves complex oxidation and intermolecular rearrangement reactions, and is considered to be one of the main reasons for quality and color changes during food processing and storage [64]. From an analysis of the effects of temperature and pressure on the retention of ascorbic acid in processed juices, it revealed that the dominant factor determining the stability of ascorbic acid is the temperature, which directly affects the degradation rate of ascorbic acid [65]. Products containing ascorbic acid, such as fruit juice, need to undergo high-temperature pasteurization in order to guarantee safety and stability. The content of ascorbic acid in fresh orange juice ranges from 25–68 mg/100 mL. Studies have shown that the retention of ascorbic acid in the product after pasteurization (90 °C, 1 min) is about 82–92% [66,67]. It was observed that the maximum temperature of the ultra-high pressure homogenization treatment at 100, 200 and 300 MPa was 45 °C, 70 °C and 94 °C, respectively, and the continuous treatment time under the maximum temperature was 0.7 s or less. The loss of ascorbic acid in the ultra-high pressure treated juice is less than that in the traditionally heat-pasteurized juice [67]. As detected, the content of ascorbic acid in guava juice was around 42.2 ± 0.01 mg/mL. After 7 days of storage at 25 and 35 °C in the dark, ascorbic acid was degraded by 23.4% and 56.4%, respectively. Its degradation is significantly reduced at 4–10 °C [68]. The use of relatively mild temperature (75 °C) for heat treatment and a storage temperature below 25 °C is optimal for maintaining the ascorbic acid content of the product [69]. The degradation of ascorbic acid during storage and heat treatment follows first-order kinetics based on a classic dynamic model [70].

The degradation or oxidation products of ascorbic acid heated at 100 °C for 2 h include furfural, 2-furoic acid, 3-hydroxy-2-pyrone and an unknown compound. Among them, furfural is one of the main degradation products of ascorbic acid, which can polymerize or combine with amino acids to form brown melanoids, causing the browning of ascorbic acid-containing juice products [62]. Furthermore, thermally oxidized ascorbic acid was identified as a potential precursor of furan; it is a possible carcinogen usually produced in some heated food products [71]. Meanwhile, natural and synthetic antioxidants, such as chlorogenic acid, have a certain mitigation effect on the formation of furan induced by heated ascorbic acid, but the mitigation effect may decrease with the increase in heating time [72]. In addition, the thermal degradation process of ascorbic acid is also affected by pH, oxygen concentration, transition metal ions and oxidases, which is a complex system. Some researchers in food science believe that oxygen saturation decreases with increasing temperature and drops to 0 at 100 °C. However, according to the Tromans and Battino model, although 100–130 °C is the minimum oxygen solubility temperature, there is still dissolved oxygen in the system [73,74]. At temperatures above 100 °C, oxygen has a greater effect on ascorbic acid degradation than temperature. Therefore, removing all oxygen including dissolved oxygen is the best way to preserve ascorbic acid at high temperatures [75].

### 3.3. Light

In addition to heat-treatment sterilization, ultraviolet radiation (240 nm–300 nm) is a promising alternative and is gradually being used more for fruit juice sterilization [76]. The ultraviolet sterilization method includes the use of high-intensity pulsed ultraviolet radiation with wavelengths between 200 and 400 nm and a monochromatic ultraviolet system of which approximately 90% of the energy comes from a single wavelength [77]. However, ascorbic acid absorbs ultraviolet radiation in the wavelength range of 229–330 nm and undergoes degradation [78]. The formation of UV-induced free radicals may accelerate the loss of ascorbic acid. Ascorbic acid continues to degrade after UV treatment; higher initial UV dose values and storage temperature accelerate the degradation of ascorbic acid in the later stage [77]. In addition, the pH of the solution also affects the photo-degradation of ascorbic acid. Under alkaline conditions, AH^-^ produced by ionization of AH^2^ is more prone to photo-degradation than AH^2^ [79]. It is worth noting that the ingredients in products may absorb or scatter UV radiation, thereby affecting the degradation of ascorbic acid. Niacinamide, as a component of vitamin B-complex with vitamin C, acts as a photo-degradation accelerator to reduce the stability of ascorbic acid under UV-irradiation [79].

## 4. Strategies for Improving the Encapsulation and Delivery of Ascorbic Acid

### 4.1. Low-Molecular-Weight Stabilizer and Derivatives

Ascorbic acid can be protected by adding other antioxidants. Food is a system in which multiple ingredients coexist, and there may be preservation effects of certain antioxidants, which involve regeneration mechanisms [80]. Ascorbic acid and flavonoids can regenerate α-tocopherol by reacting with α-tocopheroxyl radical. The bond dissociation energy of coexisting antioxidants that play a regenerative effect is lower than or close to the O-H bond [81]. Similarly, ascorbic acid can also be regenerated by certain antioxidants. It is well known that the conversion between ascorbic acid and its degradation product dehydroascorbic acid is reversible. Tert-butyl hydroquinone (TBHQ), which is often used as an antioxidant in high-fat foods, has been found to accelerate the conversion of dehydroascorbic acid to ascorbic acid, thereby stabilizing ascorbic acid. This reaction follows the first-order kinetic model, and the regeneration efficiency is proportional to the reaction time [82]. Moreover, glutathione with the free sulfhydryl group acts as a nucleophile and reducing agent. In the ascorbic acid solution, glutathione reduces dehydroascorbic acid and inhibits the degradation of ascorbic acid. The degradation kinetic model of ascorbic acid gradually changes from first-order to zero-order with the increase in glutathione concentration [56]. Meanwhile, as an effective antioxidant, ferulic acid has a synergistic effect with ascorbic acid. The oxidation–reduction potential of ferulic acid (0.595) is significantly higher than that of ascorbic acid (0.282), thus the former protect effect on ascorbic acid is indirect. There is a hypothesis that ferulic acid preferentially reacts with pro-oxidant intermediates or acts as a sacrificial substrate [12]. As mentioned above, low-molecular-weight stabilizers can inhibit the degradation of ascorbic acid to a certain extent, but it is hard to mask the acidic taste of ascorbic acid.

Considering the long-term mechanism of antioxidation and the high stability requirements of commercial products, ascorbic acid derivatives are also widely used, in addition to adding antioxidants or preservatives to stabilize ascorbic acid. For example, 2-O-D-glucopyranosyl-L-ascorbic acid, the glycosylated ascorbic acid in which the hydroxyl group on the C_2_ position is substituted by glucose residue, has excellent thermal stability and antioxidant properties [83]. Its application in anthocyanin-containing beverages can avoid the degradation of anthocyanins and maintain a high level of vitamin C content [84]. Ascorbate derivatives are also formed by introducing a phosphate group or combining sodium and magnesium salts at the C_2_ position of ascorbic acid, showing better stability than ascorbic acid [85]. In addition to hydrophilic ascorbic acid derivatives, there are lipophilic-derivatives such as ascorbic acid 6-palmitate and tetra-isopalmitoyl ascorbic acid. However, these derivatives need to undergo some reactions in vivo to be converted into ascorbic acid and exert their physiological activities, and the high-cost is a limitation of their application into large-scale commercial products.

### 4.2. Construction of Carriers Based on Bio-Macromolecules

#### 4.2.1. Chemical Interaction

Several technologies have been widely used to construct biomacromolecule-based carriers for ascorbic acid (Table 1), in order to shield the unfavorable environmental factors and improve the taste of the product. These processes involve physical and chemical interactions between carriers and ascorbic acid; chemical interactions mainly refer to covalent and non-covalent bonds.

Proteins are generally recognized as safe (GRAS) and have high nutritional value. The delivery systems based on proteins have received widespread attention in food field due to their biocompatibility, biodegradability and tunability. Ascorbic acid binds to β-lactoglobulin (β-LG) through ion contact to form a more stable conjugate than human serum albumin (HSA) and bovine serum albumin (BSA). β-LG, HSA and BSA can, respectively, bind about 50–60%, 40–55% and 35–50% of ascorbic acid, and the proteins can be used to deliver vitamin C in vitro [86]. Through a cationization reaction, the quaternary ammonium salt cationic group was attached to the soybean protein isolate (SPI) chain, which increases the solubility of the protein and favors the encapsulation of ascorbic acid [87]. However, the low loading capacity and carrier instability in the stomach and intestines are the main challenges that restrict proteins from being ideal delivery vehicles for ascorbic acid. Since the excellent hydrophilicity of ascorbic acid and its same charge as most proteins (isoelectric point, pI < 7) at physiological pH, their interactions such as hydrophobic interaction, electrostatic interaction, hydrogen bonds and van der Waals forces are usually weak or absent. This results in a low encapsulation efficiency and rapid release of ascorbic acid from protein nanoparticles in the aqueous solutions.

Chitosan is a cationic polysaccharide with excellent chelating and cross-linking properties and is widely used as a delivery vehicle in the food field. The formation of chitosan nanoparticles requires cross-linking with polyanions, such as tripolyphosphate (TPP). The amino groups of chitosan in the polymer backbone can interact with ascorbic acid to form a strong hydrogen bond, which captures and retains ascorbic acid on the polysaccharide [10,88]. The formed chitosan-ascorbic acid complexes have high singlet oxygen scavenging ability and then maintain the high antioxidant capacity of ascorbic acid. The nanoscale size and positive charge of the particles are very important for their adsorption on the mucosa, which is conducive to achieving a high uptake rate of the loaded ascorbic acid by the intestinal cells. Chitosan-ascorbic acid complex nanoparticles increase the residence time of ascorbic acid in the digestive tract of trout. Compared with protein nanoparticles, chitosan nanoparticles strengthen the interaction with ascorbic acid through electrostatic interaction, but the encapsulation efficiency is still relatively low [11]. This is related to the molecular weight and concentration of chitosan, the addition of ascorbic acid and the measurement method of the encapsulation. There are two views about the influence of chitosan molecular weight on the encapsulation efficiency of ascorbic acid. One is that high-molecular-weight chitosan has more surface charges to bind with more ascorbic acid molecules, thus the long backbone can capture more ascorbic acid. As the molecular weight of chitosan increased from 65 kDa to 110 kDa, the content of ascorbic acid loaded increased from 30% to 70%, respectively. With the further increase in chitosan molecular weight, the particle size increases but the overall surface area decreases, resulting in a decrease in the encapsulation efficiency of ascorbic acid [16]. Short fragments of low-molecular-weight chitosan are easier to protonate free amino groups, thereby complexing with ascorbic acid through electrostatic interactions. The average diameter of 55-kDa chitosan complex particles is 70.6 nm, and the loading efficiency of ascorbic acidic is about 66% [89].

#### 4.2.2. Physical Barrier

In order to maintain the stability of ascorbic acid in food applications, ascorbic acid can be loaded into biomacromolecule-based delivery vehicles through physical encapsulation and adsorption. Compared with ascorbic acid nanoparticles chelated with protein and chitosan, the construction of physical barriers such as microcapsules based on protein and polysaccharide, solid lipids and liquid state multiple emulsions have a better loading capacity of ascorbic acid in the core and hence, this improves stability.

The process of encapsulating ascorbic acid in a core walled by polymers coating to isolate it from the external adverse factors is microencapsulation. The current preparation methods of microcapsules mainly include spray chilling, spray drying and complex coacervation. Among them, spray drying is one of the most common techniques due to its low cost, continuity and easy industrial scale production [90]. The selection of wall materials includes various proteins and polysaccharides, such as gum Arabic, maltodextrin, pectin, xyloglucan, sodium alginate. Gum Arabic and sodium alginate are low-cost and GRAS category polysaccharides, which are often used as food additives. The sodium alginate/gum Arabic microcapsules prepared by spray drying have an excellent loading capacity of ascorbic acid, which can reach more than 90%. Meanwhile, the thermal stability temperature of ascorbic acid is increased to 188 °C, which is higher than the temperature required for product preparation [91]. The xyloglucan extracted from *Hymenaea courbaril var. courbaril* seeds is a water-soluble polysaccharide containing gum Arabic, which is used as a thickener, stabilizer and crystallization inhibitor in the food industry. The spray-dried microcapsules can encapsulate around 96% of ascorbic acid. The system shows strong antioxidant activity and inhibits the formation of furan, an ascorbic acid degradation product, during the preheated process of products. After 60 days of storage at room temperature, the retention of ascorbic acid in the system is still around 90% [92]. However, the high viscosity of high-concentration polymers limits the granulation by spray-drying. To a certain extent, the loading capacity is related to the wall-to-core ratio and increases with the increase in the coating of wall materials [15]. Complex coacervation is the phase separation of at least two hydrocolloids from the initial solution, and then the coacervate phase is deposited around the suspended or emulsified bioactive compounds. One of the hydrocolloids is in the colloidal state. On the contrary to hydrophobic bioactive compounds, hydrophilic ascorbic acid needs to be emulsified before it is prepared [93]. Compared with spray drying, this method does not involve a heat treatment process and is more suitable for encapsulating thermally unstable ascorbic acid [94]. The microcapsules prepared with gelatin and pectin as wall materials improve the thermal stability of ascorbic acid, although the solubility of the microcapsules is relatively low [15]. The encapsulation efficiency of ascorbic acid using gelatin and acacia as wall materials is about 97% [93].

The systems based on lipids, such as solid lipid microcapsules and emulsions, can be obtained by high-pressure homogenization, microfluidics, and solvent evaporation. The solid lipid microcapsules prepared by polyglyceryl monostearate (PGMS) have the encapsulation capacity of ascorbic acid up to about 94%. The system can be added in to fortify milk, significantly inhibiting the Maillard reaction between milk proteins and ascorbic acid. Sensory analysis showed that there was no significant difference in most aspects between the control sample and the fortified sample encapsulated with ascorbic acid after 5 days of storage [95]. As reported, palm fat was used as wall material to fabricate the solid lipid microcapsules to encapsulate and protect ascorbic acid using a microfluidic technique. The internal phase was added with salt or chitosan to further improve the encapsulation efficiency of ascorbic acid. The two different mechanisms involve pore blockage and ascorbic acid chelation [6]. This system has better physical isolation performance than protein and/or polysaccharide solid microcapsules. However, the operation process includes thermal melting and ice bath cooling of liposomes. This method is limited to the laboratory scale and is difficult to industrialize. On the other hand, the storage stability of ascorbic acid in oil-containing systems may be affected by lipid oxidation and thermodynamic instability of emulsions, which is lower than that of carrier-stable protein and polysaccharide microcapsule systems [96,97].

The microcapsule system based on the physical barrier has a better loading capacity of ascorbic acid than complex nanoparticles (Table 1). In addition to the properties of the delivery carriers, it may also be related to the different measurement method of encapsulation efficiency. For delivery systems in micro-scale, the measurement conditions for encapsulation efficiency of ascorbic acid are gentler than those of protein and/or polysaccharide nanoparticles. The determination method includes separation by standing, ultrasonic and filter paper filtration [82,83,84,85,86]. Compared with the ultra-isolation method [79,80] used in the nanoparticle system, these methods reduce the release and diffusion of ascorbic acid during the measurement process.

**Table 1:** Different types of bio-macromolecule delivery vehicles of ascorbic acid.

Carrier	Material	Technology	Protective Effect	Encapsulation Efficiency	Reference
Microcapsules	Sodium alginate/gum Arabic	Spray drying	Thermal stability temperature of ascorbic acid is increased to 188 °C.	>90%	[91]
	Xyloglucan	Spray drying	After 60 days of storage at room temperature, the retention of ascorbic acid is around 90%.	~96%	[92]
	Gum Arabic/rice starch	Spray drying	The retention of ascorbic acid is around 81.3% after 90 days of storage at 21 °C.	~99.7%	[98]
	Gelatin/pectin	Complex coacervation	With low hygroscopicity and high thermal stability.	23.7% to 94.3%	[15]
	Gelatin/acacia	Complex coacervation	The retention of ascorbic acid is around 44% and 80% after 30 days of storage at 37 °C and 20 °C, respectively.	≥97%	[93]
Liposome	Palm fat/chitosan	Microfluidic technique	After 30 days, retained 98.58% and 97.62% of ascorbic acid at 4 °C and 20 °C, respectively.	~ 96.6%	[6]
	Polyglyceryl monostearate	Spray chilling	The system can inhibit the Maillard reaction between milk proteins and ascorbic acid.	~94.2%	[98]
	Milk fat globule membrane-derived phospholipids	Microfluidic technique	After 7 weeks at 4 °C and 25 °C, ascorbic acid in liposomes retained 67% and 30%, respectively.	~26%	[97]
W/O/W emulsions	Gelatin/tetraglycerin monolaurate condensed ricinoleic acid ester/decaglycerol monolaurate	Homogenization	The half-life for W/O/W emulsions containing 30% ascorbic acid at 4 °C was about 24 days.	≥90%	[99]
	Soybean oil/tetraglycerin condensed ricinoleic acid ester/gelatin	Homogenization and microchannel emulsification	The ascorbic acid exhibited 80% retention after 10 days storage at 4 °C.	>85%	[100]

#### 4.2.3. Controlled Release of Ascorbic Acid

The challenge of ascorbic acid in food applications is not only to maintain its stability, but also to improve the effectiveness of delivery it to the active site. The release of bioactive compounds in the body is expected to occur in the intestine rather than the stomach, because the absorption mainly occurs in the small intestine. It was found that ascorbic acid in pomegranate juice was approximately 29% degraded during gastric digestion, which severely reduced the bioavailability of ascorbic acid. Additionally, the compounds that are transported by specialized processes are usually only absorbed in certain parts of the gastrointestinal tract. The absorption of riboflavin begins in the upper region of the small intestine, as does ascorbic acid [101]. Therefore, the changes in gastric and intestinal transit rates may affect the absorption efficiency of orally administered bioactive compounds. Additionally, the bioavailability of oral ascorbic acid is related to the following key steps: (1) Release of ascorbic acid in the gastrointestinal tract, and its solubility in gastrointestinal fluids. (2) Intestinal epithelial cells absorb ascorbic acid and undergo biochemical transformation. Studies have shown that a single high dose of ascorbic acid causes a temporary increase in plasma that is rapidly absorbed by the gastrointestinal tract and then quickly excreted in the urine [102]. A form of ascorbic acid that can be slowly released in the intestine is desired to maintain a constant level of ascorbic acid in plasma.

The release process of encapsulated ascorbic acid is as follows: absorption of solvent by the carrier, dissolution of the wall-coating, and the diffusion of inner core. The release of bioactive compounds in carriers depends on many factors, such as the selection of the wall material, the ratio of wall/core, the size of the carrier, the solubility of the bioactive compound, and the release conditions [15,97]. Ascorbic acid releases kinetics from ascorbate gummies, which were investigated using an in vitro simulated digestion model. The results show that the disintegration time of ascorbic acid candy was about 22 min, after which the functional ingredient ascorbic acid was gradually released, reaching 93.6% within 2 h. Notably, the components in gastric juice may have an effect on the release of ascorbic acid, with gastric juice containing 5% starch slowing the release of bioactive ascorbic acid in the gummies, but other dietary components had no significant effect on its release [103]. This may be related to the encapsulation of ascorbic acid in starch in the stomach. Compared with the afore-mentioned delivery vehicles based on polysaccharide and lipid, the protein carrier has poor stability in the stomach. The low pH of the gastric environment and the presence of pepsin cause the denaturation and degradation of the protein carrier, leading to the leakage of loaded bioactive compounds in the stomach before reaching the small intestine [104]. Gelatin/pectin microcapsules show a high loading capacity of ascorbic acid. However, due to the dissolution of gelatin coating in the gastric environment, the release of ascorbic acid in the gastric environment is faster than in the intestine [15]. Therefore, it is necessary to design a carrier that is relatively stable in the stomach and which can provide a sustained release of ascorbic acid in the intestine.

The small size and positive charge of the particles contribute to the high uptake rate by intestinal cells. The loading in chitosan nanoparticles effectively prolong the residence time of ascorbic acid in the intestine of rainbow trout [16]. Nanoparticles based on chitosan with low molecular weight have a higher delivery rate of ascorbic acid. The mechanism of ascorbic acid released from nanoparticles in the gastric environment and the intestinal environment are diffusion and erosion, respectively. Under the neutral conditions of the intestine, the ion exchange between chitosan and the release medium leads to the erosion of nanoparticles. The release rate of ascorbic acid increased from 30% in the stomach to more than 75% in the intestine [16]. As reported, the water-soluble derivative N,N,N-trimethylchitosan (TMC) as a carrier can efficiently transport hydrophilic molecules through mucosal epithelial tissues such as the oral cavity, nasal cavity, lungs and intestines [88]. Thus, the carriers based on chitosan coatings may be an effective strategy to achieve intestinal release of ascorbic acid. Based on the continuous deposition of positively charged chitosan and negatively charged sodium alginate on the surface of anionic nano-liposomes, a liposomal polyelectrolyte delivery system of ascorbic acid was prepared. The clinical results showed that the bioavailability of orally administered liposomal ascorbic acid was 1.77 times higher than that of non-liposomal ascorbic acid, with higher bioavailability [105]. The ability of the outer layer of chitosan to withstand the gastric environment is beneficial for maintaining the stability of the carrier structure. The excellent sealing properties of liposomes and better penetration with enterocyte phospholipid bilayers also contributed to the improved bioavailability of released ascorbic acid.

## 5. Commercial Application of Ascorbic Acid

Based on the afore-mentioned biological activity of ascorbic acid, ascorbic acid is mainly used as an antioxidant to inhibit food browning and as a dietary supplement for humans. Ascorbic acid is mainly used as an antioxidant to protect the senses of foods. As is well known, polyphenol oxidase catalyzes the enzymatic browning of phenol substrates to yield dark-colored melanin. Browning affects product sensory qualities and reduces consumer acceptance. Adding xyloglucan microcapsules containing ascorbic acid to baked foods such as tilapia fish burgers can significantly inhibit the browning that occurs during the preparation process and maintain the sensory qualities of the product [93]. The chitosan/tripolyphosphate nano-aggregates containing ascorbic acid enhanced the inhibition of mushroom slices browning induced by tyrosinase [106]. Acute heat stress during transport is known to predispose rainbow trout quality to deterioration, with negative effects on the histological, physicochemical and microbiological quality of fillets. Treatment with added ascorbic acid partially mitigated damage caused by acute heat stress. It can maintain tissue structure, delay protein oxidation and then prolong the shelf life of fish fillets to about 2 days [107]. In addition, as a nutritional supplement, ascorbic acid plays an important role as a co-factor in many biological processes. Unfortunately, fishes lack L-gluconolactone oxidase and cannot biosynthesize ascorbic acid by themselves, which is not conducive to the growth of their bone matrix and connective tissue. Lack of ascorbic acid can cause reduced wound-healing capacity and bone deformities in fish [108]. At present, in the aquaculture area, ascorbic acid is widely added to fish diets. Based on the healthcare function of ascorbic acid, it is also vital in nutrition fortification products. As an important source of protein supplementation, dairy products are popular beverages all over the world. At present, milk and soymilk have been fortified with ascorbic acid, including ascorbate and ascorbic acid isomers, to improve the iron absorption in the small intestine [109,110].

Food fortification can improve micronutrient malnutrition. It is worth noting that a category of foods tailored according to the necessary nutrients for a healthy life and their specific concentrations and ratios are called designer foods, also known as health foods, and are sought after and recognized by consumers. Such products often contain a variety of bioactive compounds. By adding calcium and antioxidants such as vitamins E and C to low-fat chicken patties, a high-quality product with high-quality animal protein, fat, multivitamins and minerals can be prepared. Ascorbic acid not only acted as a nutritional additive, but also maintained better color and flavor of chicken patties, and inhibited the formation of nitrosamines in the meat [111]. The addition of sodium ascorbate and vitamin A to pig feed can significantly improve the growth performance, antioxidant capacity and immune function of weaned piglets. Meanwhile, as an antioxidant, sodium ascorbate can delay the degradation of vitamin A [112]. A cornstarch-based baking premix was developed by addition of vitamin B, vitamin C and digestible iron, zinc, selenium and iodine. Although the added ascorbic acid in the baked bread degraded due to high temperature, it strengthened the structure of the bread and was benefit to product quality [113]. Meanwhile, it was found that the combination of butylated hydroxytoluene and ascorbic acid significantly inhibited the oxidation and isomerization of vitamin A in skim milk powder during thermally accelerated storage [104].

There are two major aspects in the current development of ascorbic acid-fortified products. On the one hand, the natural ascorbic acid is directly added, in order to use its antioxidant activity to maintain the sensory appearance of the product during the shelf life. The cost is low, but the retention activity of the final product is low. Another aspect is the addition of ascorbic acid derivatives, which is to ensure that sufficient physiological activity can be expressed after ingestion of the product. However, the cost of ascorbic acid derivatives is high, and they need to be converted before they can exert their functional properties. The related products of ascorbic acid and its main derivatives in the food fields in recent years are summarized in Table 2. Although various delivery technologies are available, they are still in the developmental stage of industrial transformation and have not been widely used. Combined with the above analysis of delivery strategies, these may be limited by the cost of wall materials, and the complexity of the process, which is not suitable for large-scale industrial production. Therefore, researchers still need to explore low-cost, simple, and high-yield encapsulation techniques of ascorbic acid for industrial application.

## 6. Conclusions

In this review, the bioactivity and stability of ascorbic acid are introduced. There are many strategies for improving the bioavailability of ascorbic acid, and the influence of delivery systems on the stability and release properties of ascorbic acid is discussed. Besides the addition of low-molecular-weight antioxidants and preservatives, encapsulation technology is more and more widely used in the food field. The stabilization mechanism includes chemical chelation and physical barrier. Since the positively charged chitosan can interact with ascorbic acid through electrostatic interaction and hydrogen bond, it is the most superior carrier material. The complex system is mostly in the form of nano-sized particles. On the other hand, biomacromolecules can construct microcapsules with coatings through a series of technologies, such as spray drying, microfluidic technique and complex coacervation. The physical barrier restricts ascorbic acid within the inner core, reducing the contact between it and the external environment. The two mechanisms have their own limitations. For example, chemically complexed nanoparticles are beneficial to the absorption by mucosal membranes, but the encapsulation efficiency of ascorbic acid is low and ascorbic acid is accessible to solvent. The coating of the microcapsules can effectively shield the inner ascorbic acid from external environment, but the operation is more complicated and requires the assistance of a variety of equipment. In addition, the larger size and poor water-solubility of the microcapsules limit the absorption in the body to a certain extent. Therefore, understanding the pro-degradation factors of ascorbic acid and its properties are conducive to the targeted design of delivery systems. Adopting low-cost methods to design an effective fortification strategy to improve the stability of ascorbic acid during processing and storage is still the focus and challenge for researchers.

## Figures and Tables

**Table 2:** Commercial products fortified with ascorbic acid and its derivatives.

Ascorbic Acid or Ascorbate	Product	Property of Added Bioactives	Challenges of Application	References
L-ascorbic acid	Liqueur chocolate, milk fortification, edible coating, juice, meat patties	With antioxidant properties and a series of physiological activities such as iron metabolism, it can eliminate bacterial biofilms and the cost is low.	Poor stability, sour taste.	[31,33,82,100,114,115,116]
L-ascorbic acid sodium	Fish feed, formulae and weaning foods, cured hams	With antioxidant properties and the cost is low.	Poor stability, and compared with ascorbic acid, sodium ascorbate has a potential anti-nutritional effect on protein after high-temperature baking.	[117,118]
2-O-D-glucopyranosyl-L-ascorbic acid	Berry beverage, black rice baking products, cured meat products, aquatic products	With anti-oxidation and stability, it avoids the degradation of anthocyanins caused by ascorbic acid and releases ascorbic acid under the catalysis of enzymes in vivo.	High cost and lowyield in industrial production.	[84,119,120]
L-ascorbic acid palmitic acid ester	Formula milk, heme iron-fortified bakery product, frying oil, nutritional powders	It is a lipophilicity L-ascorbic acid esters derivatives with antioxidant properties and can be converted into ascorbic acid by esterase.	The thermal stability is poor, and chemically modified products often contain mixtures.	[121,122,123,124]

## Abbreviations

RDA	recommended dietary allowances
DHA	dehydroascorbic acid
ROS	reactive oxygen species
O_2_·^−^	superoxide radicals
^1^O_2_	singlet oxygen
H_2_O_2_	hydrogen peroxide
OH·	reactive hydroxyl radicals
AH_2_	ascorbic acid
AH^−^	ascorbate
A^2^^−^	ascorbate dianion
TBHQ	tert-butyl hydroquinone
GRAS	generally recognized as safe
β-LG	β-lactoglobulin
HAS	human serum albumin
BSA	bovine serum albumin
SPI	soy protein isolate
TPP	tripolyphosphate
PGMS	polyglyceryl monostearate
TMC	N,N,N-trimethylchitosan
